# Dietary luteolin attenuates chronic liver injury induced by mercuric chloride via the Nrf2/NF-κB/P53 signaling pathway in rats

**DOI:** 10.18632/oncotarget.17334

**Published:** 2017-04-21

**Authors:** Haili Zhang, Xiao Tan, Daqian Yang, Jingjing Lu, Biying Liu, Ruiqi Baiyun, Zhigang Zhang

**Affiliations:** ^1^ College of Veterinary Medicine, Northeast Agricultural University, Harbin, 150030, China

**Keywords:** luteolin, HgCl_2_, hepatotoxicity, Nrf2, NF-κB

## Abstract

Mercury exposure is a common cause of metal poisoning which is biotransformed to highly toxic metabolites thus eliciting biochemical alterations and oxidative stress. Luteolin, a phenolic compound found in many natural products, has multiple biological functions. Our study was aimed to explore the biological effects of luteolin in a liver injury model induced in rats by mercuric chloride (HgCl_2_). Criteria for injury included liver enzyme, glutathione and malondialdehyde levels, histopathology, TUNEL assay, hepatocyte viability and reactive oxygen species levels. The results showed that luteolin protected against HgCl_2_-induced liver injury. Luteolin increased total nuclear factor-erythroid-2-related factor 2 (Nrf2) levels in the presence of HgCl_2_. Upregulation of its downstream factors, heme oxygenase-1 and NAD(P)H quinone oxidoreductase 1, was also observed. This suggested that protection by luteolin against HgCl_2_-induced liver injury involved Nrf2 pathway activation. Luteolin also decreased expression of nuclear factor-κB (NF-κB) and P53. HgCl_2_ exposure led to increased Bcl-associated X protein (Bax), and decreased Bcl-2-related protein long form of Bcl-x (Bcl-xL) and B-cell leukemia/lymphoma-2 (Bcl-2) expression, leading to an increased Bax/Bcl-2 ratio. Taken together, our data suggested that decreasing oxidative stress is a protective mechanism of luteolin against development of HgCl2-induced liver injury, through the Nrf2/NF-κB/P53 signaling pathway in rats.

## INTRODUCTION

Mercury, in the inorganic form mercuric chloride (HgCl_2_), is the third most dangerous heavy metal and metalloid element, after arsenic and lead, according to the Agency for Toxic Substance and Disease Registry [1]. Although mercury has long been considered a toxic metal, it still has numerous important industrial uses and poisoning from occupational exposure and environmental pollution continues to be a concern [2]. In several *in vitro* and *in vivo* studies, damaging effects induced by mercury were related to adverse health impacts including cancer, neurological disorders and cardiovascular diseases [3–4]. Among organs, the liver is the major site for handling toxins, with a central role in physiological metabolism and various detoxification reactions. Primary murine hepatocytes are frequently used as a model for investigating the toxicity and protective mechanisms associated with various toxins. Based on available experimental data, it is a reasonable hypothesis that mercury toxicity involves oxidative stress, inflammation and apoptosis.

In previous reports, treatments for mercury exposure frequently included the dithiol chelators, meso-2,3-dimercaptosuccinic acid (DMSA) and 2,3-dimercaptopropanesulfonic acid (DMPS), which were shown to increase mercury excretion and relieve symptoms [5]. However, these drugs have an appreciable risk of side effects [6]. We previously described remarkable effects of some natural products on prevention and treatment of arsenic poisoning [7–9]. Therefore, as rich sources of pharmaceutical compounds with low toxicity, such natural products might lead to discovery of new drugs for treating mercury poisoning.

Luteolin (Lut, 3′, 4′, 5, 7-tetrahydroxyflavone), a key member of the flavones, is a naturally occurring polyphenolic compound, abundant in vegetables, fruits and natural Chinese herbal medicines such as celery, grapes and peppermint. In current research, antioxidant activity generally measured by free radical scavenging assays [10–12]. Luteolin exhibited a number of biological effects, including anti-inflammatory and anti-oxidative properties, as well as anti-proliferative activities against various cancer cells [13–15]. However, to our knowledge, there have been no systematic empirical reports addressing the impact of luteolin on HgCl_2_-induced chronic hepatotoxicity.

Based on this background, our study aimed to evaluate liver toxicity of HgCl_2_ in rats, including serum biochemical parameters, oxidative stress indices and histopathologic alterations. A further goal was to define the detailed mechanisms of luteolin's action against chronic mercury intoxication in rats.

## RESULTS

### Hematological analysis

Effects of HgCl_2_ and/or luteolin treatment on various hematological attributes are summarized in Table 1.

**Table 1:** Effects of the luteolin on erythrocytes, hematocrit, hemoglobin, MCH, MCHC, MCV, platelets and leuko cyte values of rats exposed to HgCl_2_

	Control	LUT	HgCl_2_	HgCl_2_ + LUT
**Erythrocyte (× 10^6^/ml)**	8.07 ± 0.93	7.70 ± 0.80	6.38 ± 0.75*	7.37 ± 1.03^#^
**Hemoglobin (g/dl)**	156.50 ± 12.56	156.25 ± 12.00	131.00 ± 11.44*	144.38 ± 12.15^#^
**Hematocrit (%)**	40.64 ± 3.01	38.89 ± 2.78	30.29 ± 3.01*	34.76 ± 2.33^#^
**MCH (pg)**	19.23 ± 1.46	19.96 ± 1.23	19.71 ± 1.97	19.96 ± 2.67
**MCHC (g/dl)**	398.50 ± 25.17	427.13 ± 22.48	404.63 ± 34.68	409.50 ± 36.75
**MCV (fl)**	48.79 ± 2.36	47.81 ± 2.29	48.81 ± 1.39	47.70 ± 1.08
**Platelets (× 10^3^/ml)**	445.50 ± 42.30	435.50 ± 40.17	334.88 ± 34.96*	383.94 ± 23.34^#^
**Leukocyte (× 10^6^/ml)**	8.48 ± 1.29	8.03 ± 0.96	11.90 ± 1.17*	8.07 ± 1.09^#^

Data are expressed as means ± SEM (*n* = 8). *P < 0.05 compared to the control group; ^#^*P* < 0.05 compared to HgCl_2_-treated group.

Values for erythrocytes, hematocrit and hemoglobin were close to control values in rats administered only luteolin. In contrast, these parameters were significantly decreased in the HgCl_2_-treated group, compared with other groups. These HgCl_2_-induced decreases were prevented in rats also administered luteolin.

Mean corpuscular volume (MCV), mean corpuscular hemoglobin (MCH) and mean corpuscular hemoglobin concentration (MCHC) were not significantly altered by the treatments.

Meanwhile, compared with the control group, leukocyte counts were significantly increased and platelet counts significantly decreased in the HgCl_2_-treated group and luteolin administration prevented this change.

### Assessment of liver function markers

**Figure 1:**
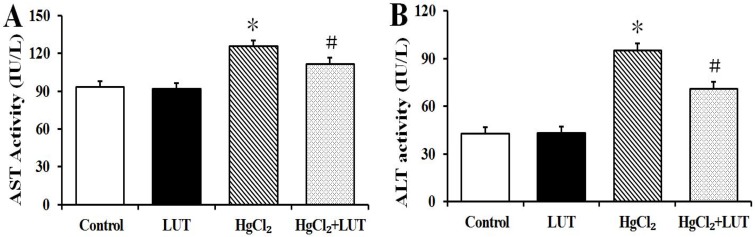
(**A** and **B**) illustrate AST and ALT activities respectively in normal and experimental groups of rats. HgCl_2_ administration increased ALP and ALT levels compared to the normal, while treatment with luteolin significantly restored this change. Data are expressed as means ± SEM (*n* = 8). *P < 0.05 compared to the control group; ^#^*P* < 0.05 compared to HgCl_2_-treated group.

Blood biochemistry was performed to evaluate the potential hepatotoxicity of HgCl_2_. Aspartate transaminase (AST) and alanine aminotransferase (ALT) are regarded as the useful markers of liver injury. Figure 1A shows that AST levels were significantly increased in the HgCl_2_-treated group. Similar results were seen with ALT (Figure 1B), indicating that HgCl_2_ induced liver injury. Luteolin administration in the HgCl_2_-treated rats led to significantly decreased activities of the two liver enzymes, compared with those treated with HgCl_2_ alone.

### Luteolin decreased oxidative stress induced by HgCl_2_ in the liver

**Figure 2:**
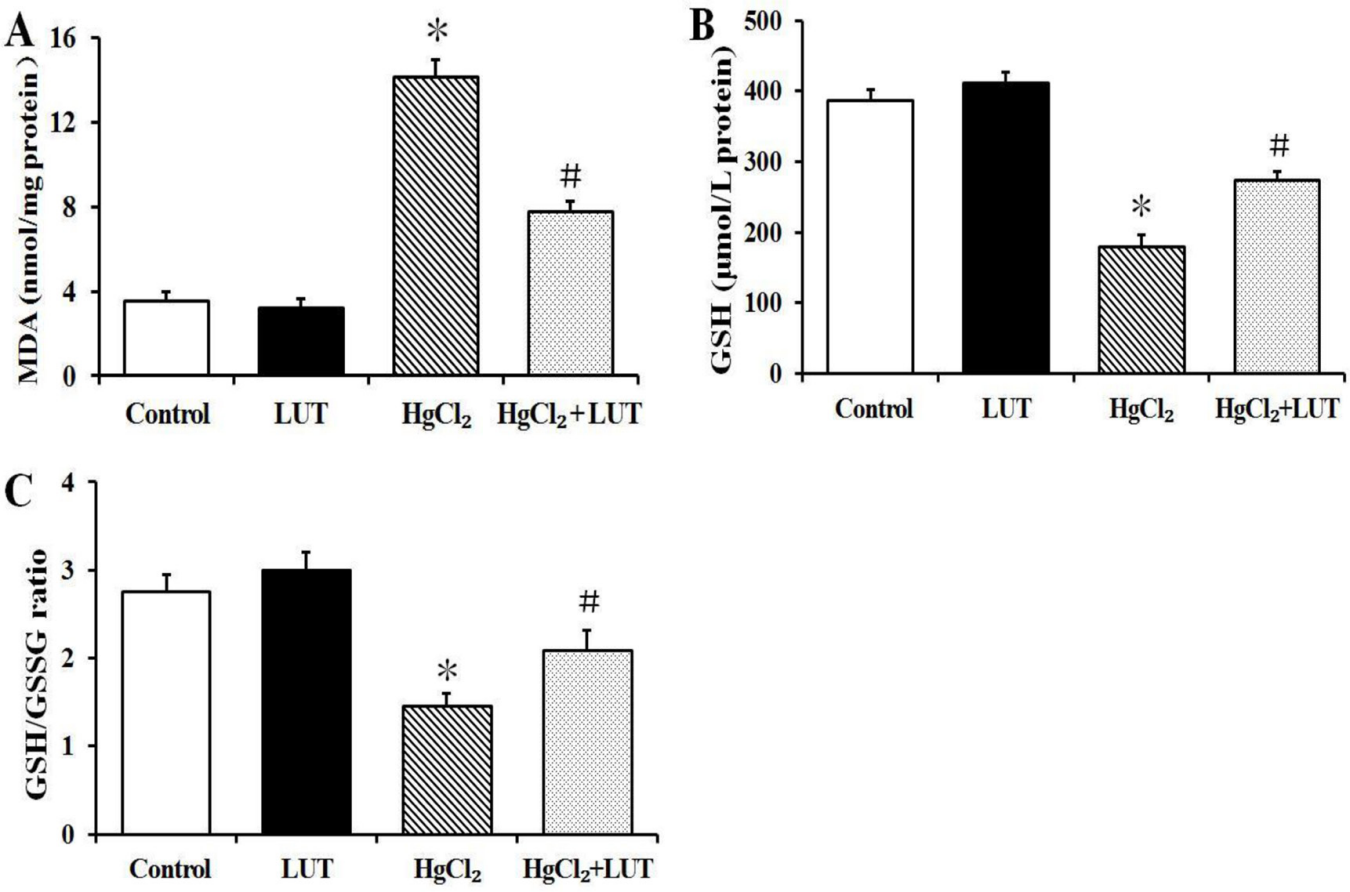
Effects of luteolin given to rats prior to HgCl_2_ administration on concentration of MDA and GSH in liver tissue Results of MDA activity (**A**) GSH activity (**B**) and GSH/GSSG ratio (**C**) in the liver are expressed as means ± SEM (*n* = 8). *P < 0.05 compared to the control group; ^#^*P* < 0.05 compared to HgCl_2_-treated group.

Various parameters relating to redox status were monitored in rats after treatment with HgCl_2_, in the presence or absence of luteolin. The results are shown in Figure 2.

Malondialdehyde (MDA) is a marker for oxidative lipid damage. MDA levels in livers of the HgCl_2_ group were greater than in the control group. Treatment with luteolin decreased liver MDA levels in rats subsequently treated with HgCl_2_ (Figure 2A).

In Figure 2B and 2C, the HgCl_2_-treated group had decreased the reduced glutathione (GSH), as well as a decreased GSH/glutathione disulfide (GSSG) ratio, compared with the control group. These effects were attenuated in the group receiving combined treatment with luteolin and HgCl_2_.

### Histopathological examination

**Figure 3:**
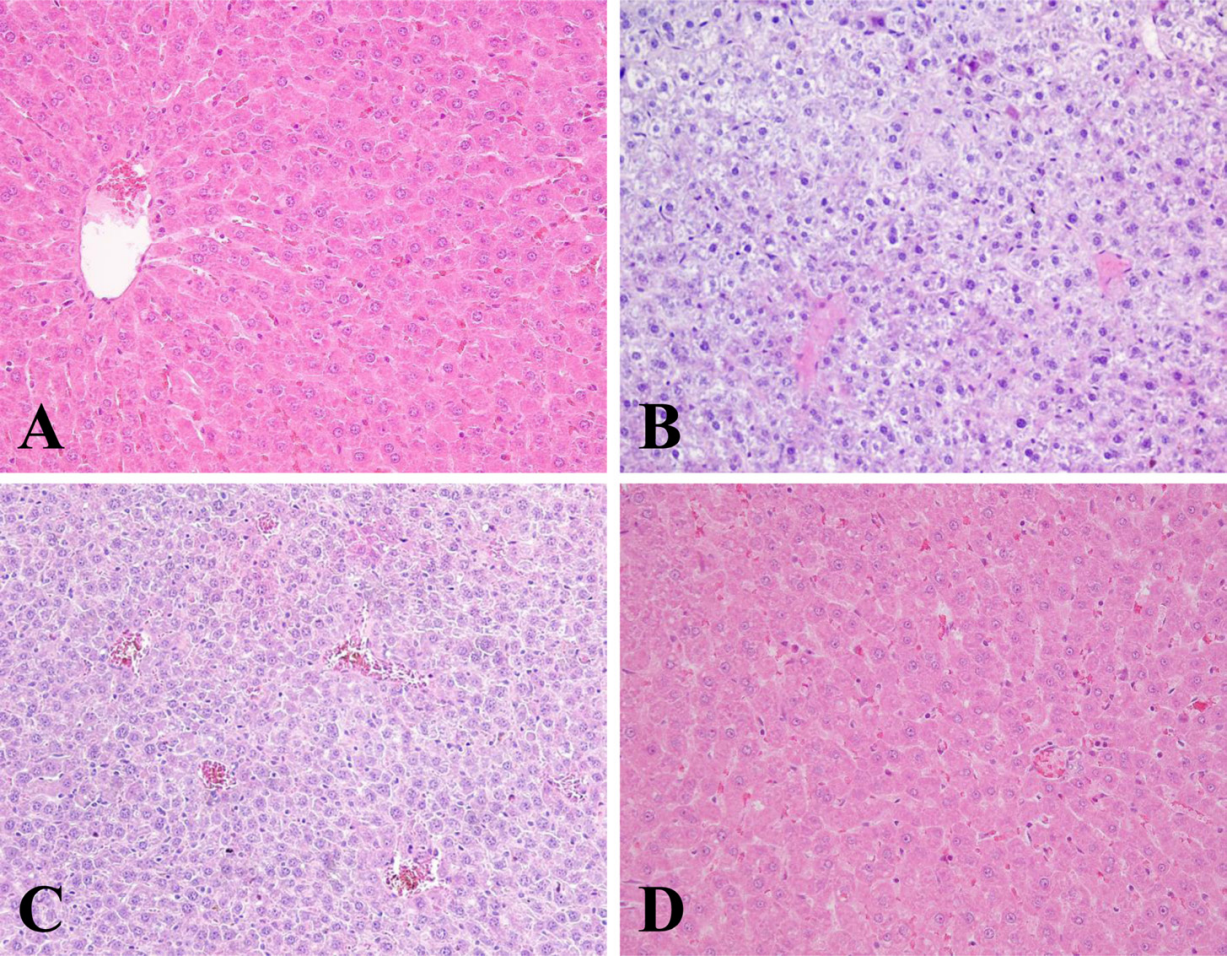
The histological of livers changed after different treatments Tissues were fixed in 4% paraformaldehyde, embedded in paraffin, and stained with H&E. Typical images were chosen from each experimental group (original magnification 200×): (**A**) control group. (**B**) Rats treated with luteolin. (**C**) Rats treated with HgCl_2_. (**D**) Rats treated with the luteolin and HgCl_2_.

We compared the histological structure among each groups using hematoxylin and eosin (H&E). In the control group, the cross-section showed hepatocytes arranged radically surrounding the central vein (Figure 3A). There were no abnormalities or histological changes observed in the livers of luteolin-treated group as illustrated (Figure 3B). HgCl_2_ administered group showed evident liver injury, which had severe hepatic necrosis, swelling, disappearing, and disarranged structure of hepatic lobules (Figure 3C). These pathological changes were attenuated in the group receiving combined treatment with luteolin and HgCl_2_, but congestion spaces and inflammatory cell infiltrating were still present (Figure 3D).

### Luteolin inhibited hepatocyte apoptosis

**Figure 4:**
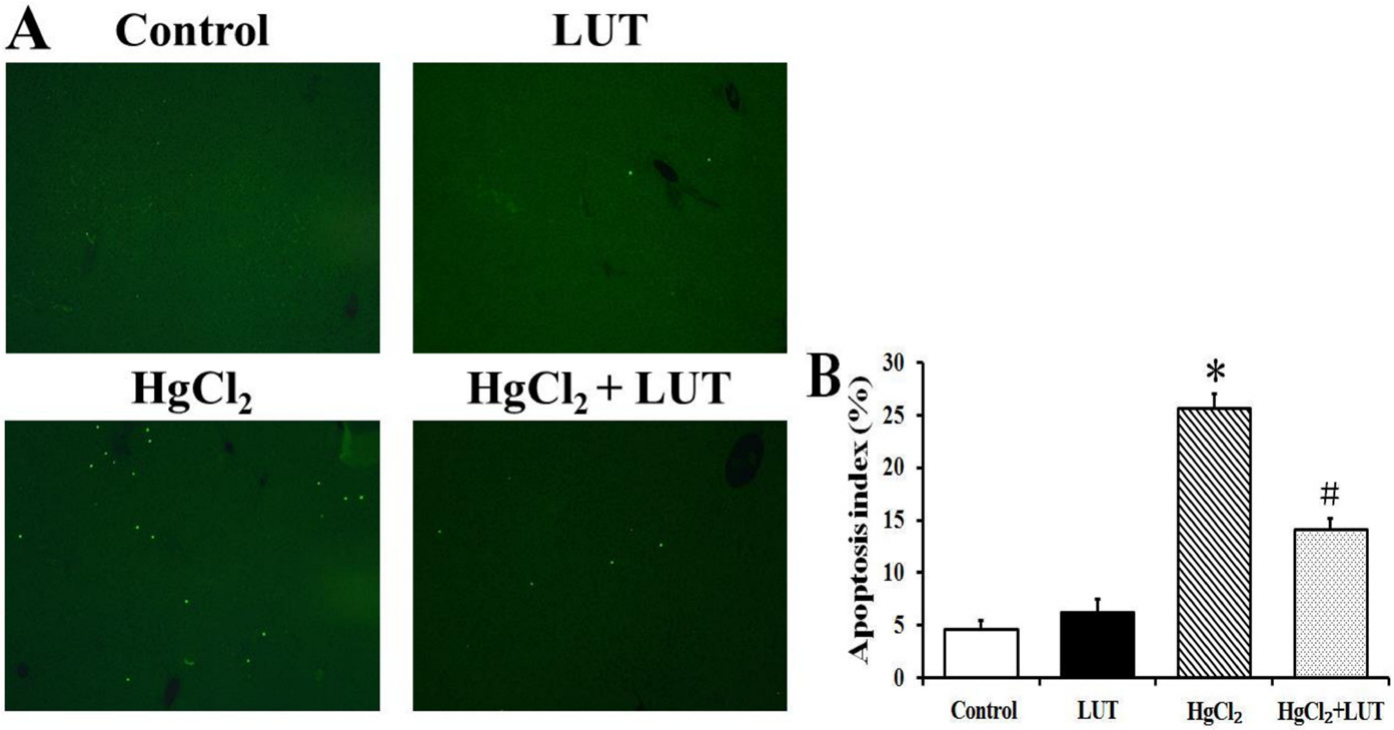
Apoptosis index was determined using TUNEL assays (**A**) Representative photographs of TUNEL staining in control, LUT, HgCl_2_ and HgCl_2_ + LUT groups. Scale bar = 20 μm. (**B**) Quantitative analysis of TUNEL-positive cells. Luteolin treatment significantly decreased the percentage of hepatocyte apoptosis after HgCl_2_ treatment. Data are expressed as means ± SEM (*n* = 8). * *P* < 0.05 compared to rhe control group; ^#^*P* < 0.05 compared to HgCl_2_-treated group.

To determine whether the heptoprotective effects of luteolin could be detected histopathologically, we employed terminal deoxynucleotidyl transferase-mediated dUTP nick end labeling (TUNEL) staining to examine apoptotic cells in the liver tissue (Figure 4). There were few TUNEL-positive cells in the control group, while HgCl_2_ induced an apoptotic index of approximate 25.73%. There was no difference in apoptosis indexes in the control group and the luteolin treated group. In HgCl_2_ treated rats, luteolin significantly decreased the apoptotic index to 14.13%.

### Luteolin prevented cytotoxicity and formation of reactive oxygen species (ROS) generation induced by HgCl_2_ *in vitro*

**Figure 5:**
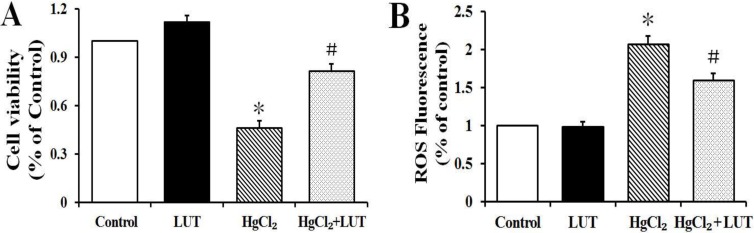
(**A**) The survival cells were determined by CCK-8 assay. (**B**) The levels of intracellular ROS in hepatocytes were determined by DCF-DA as described in Material and Methods. The results are expressed in percentage of control and presented as the means ± SEM (*n* = 6). *P < 0.05 compared to the control group; ^#^*P* < 0.05 compared to HgCl_2_-treated group.

We investigated whether luteolin could prevent HgCl_2_-induced hepatotoxicity *in vitro*. As shown in Figure 5A, hepatocytes incubated with 5 μM HgCl_2_ had decreased cell viability and this was prevented by luteolin.

We examined effects of luteolin on intracellular ROS production by a fluorescence based method. Hepatocytes were incubated with DCFH-DA and analyzed by fluorescence microscopy. As shown in Figure 5B, HgCl_2_ treatment significantly increased intracellular ROS levels, while this effect was lower in the presence of luteolin. There was no significant difference in fluorescence between the control and luteolin-treated groups.

### Effects of luteolin on forkhead box O3 (FoxO3) gene expression

**Figure 6:**
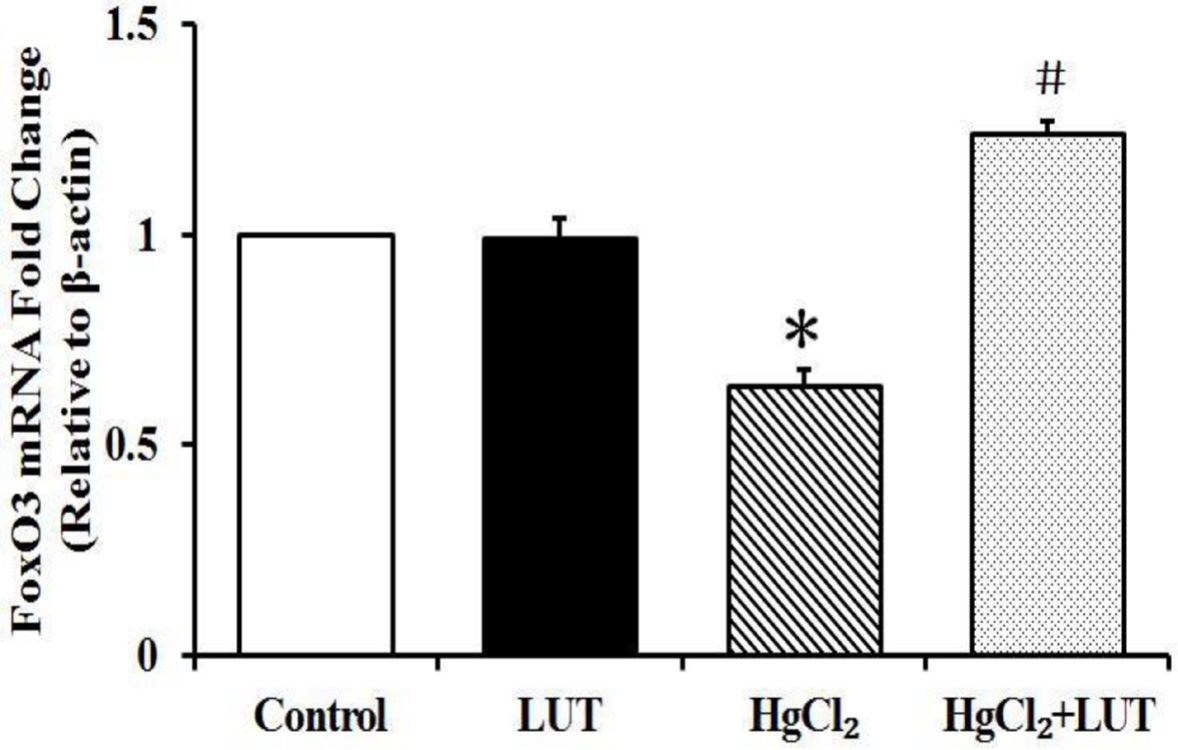
RT-PCR analysis of FoxO3 gene expression levels after HgCl2 treatment and/or luteolin and β-actin was used as an internal control Data are expressed as means ± SEM (*n* = 4). *P < 0.05 compared to the control group; ^#^*P* < 0.05 compared to HgCl_2_-treated group.

RT-PCR results indicated that FoxO3 mRNA was down-regulated after HgCl_2_-induced hepatotoxicity. Additionally, luteolin administration enhanced mRNA expression of FoxO3 compared with the HgCl_2_-treated group (Figure 6).

### Effects of luteolin on total nuclear factor-erythroid-2-related factor 2 (Nrf2) protein and Nrf2-related protein levels in the liver

To investigate effects of luteolin on Nrf2, the primary transcription factor regulating oxidative stress, we measured Nrf2 activation.

**Figure 7:**
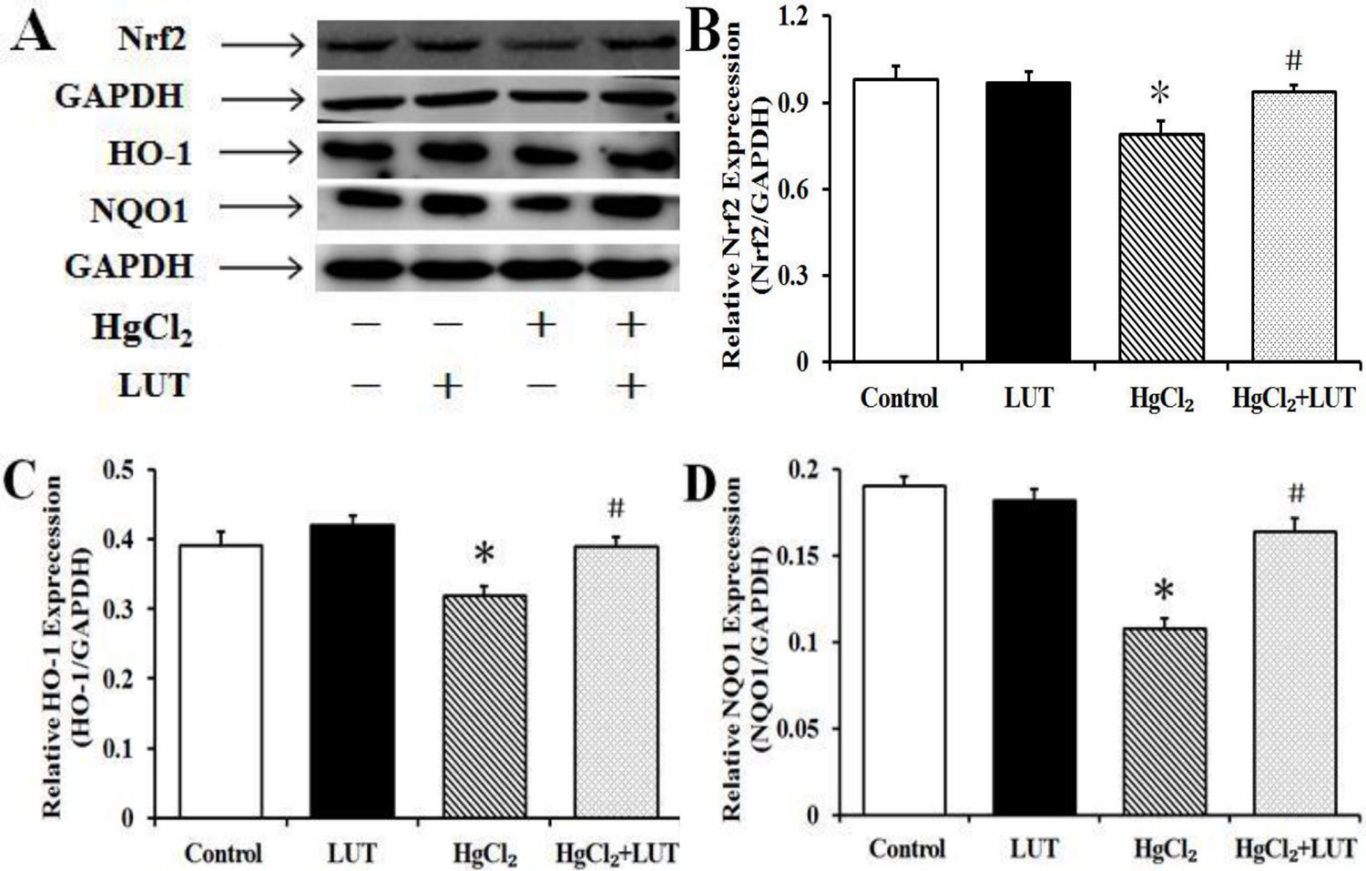
Treatment with luteolin activated the Nrf2 pathway and protected the liver against HgCl_2_-induced injury (**A**) The protein levels of Nrf2, HO-1 and NQO1. (**B**, **C** and **D**) Quantifed protein levels were shown. GAPDH was used as an internal control. Data are expressed as means ± SEM (*n* = 4). *P < 0.05 compared to the control group; ^#^*P* < 0.05 compared to the HgCl_2_-treated group.

As shown in Figure 7A and 7B, there was substantial down-regulation of Nrf2 protein expression in the HgCl_2_-treated group, compared with rats receiving no treatment or only luteolin. In addition, Nrf2 protein expression was increased by treatment with both HgCl_2_ and luteolin.

NAD(P)H quinone oxidoreductase 1 (NQO1) and heme oxygenase-1 (HO-1) are important cellular antioxidant enzymes, as well as being Nrf2-regulated genes. HgCl_2_ decreased NQO1 and HO-1 protein expression, while luteolin significantly increased levels of both proteins when administered after the HgCl_2_ (Figure 7A, 7C and 7D).

### Effects of luteolin on the mitochondrial pathway of apoptosis

**Figure 8:**
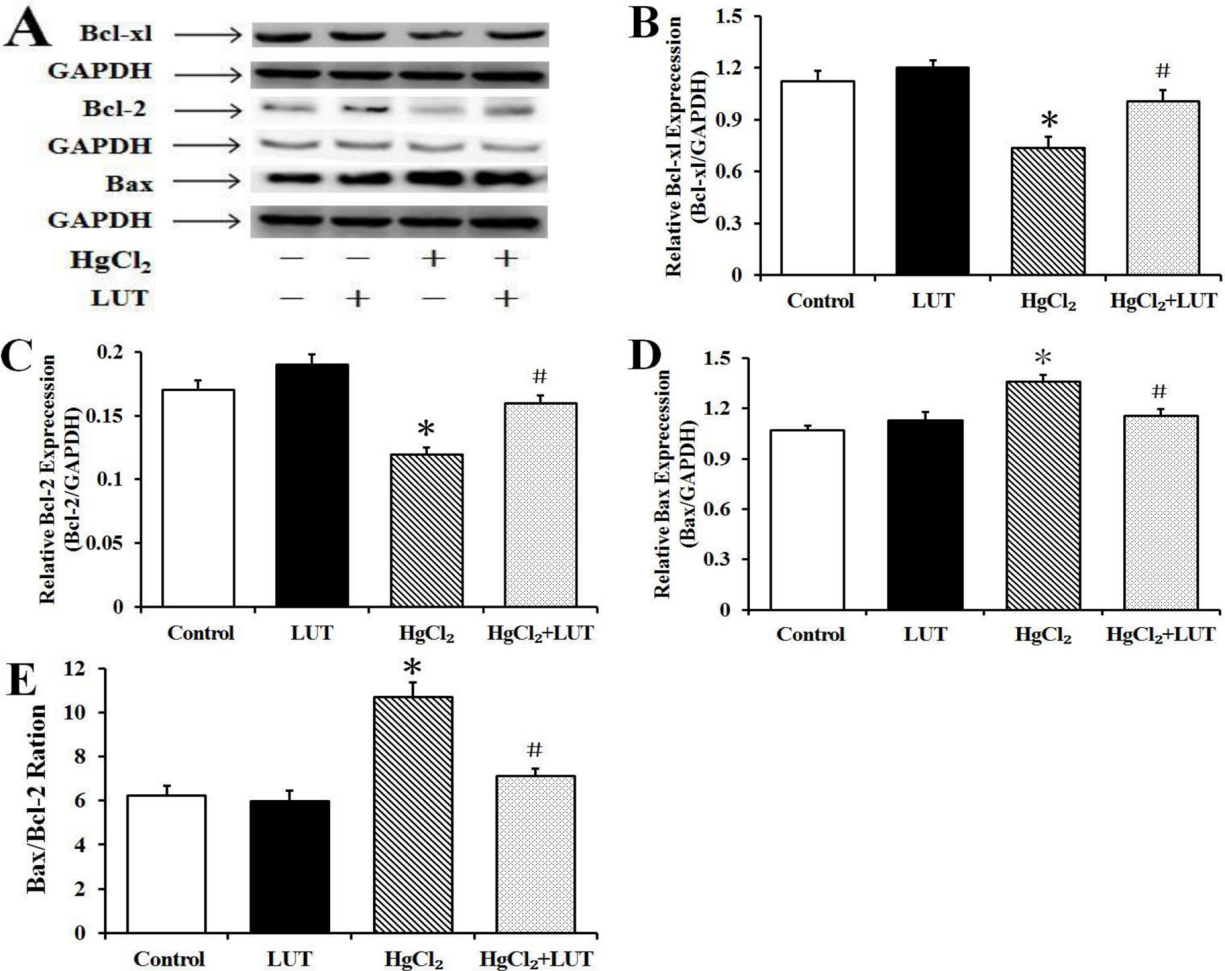
(**A**) Western blot to evaluate the expression levels of Bcl-xl (**B**), Bcl-2 (**C**), Bax (**D**) and Bax/Bcl-2 ratio (**E**) in liver tissue and gray value analysis. GAPDH was used as an internal control and data are expressed as means ± SEM (*n* = 4). *P < 0.05 compared to the control group; ^#^*P* < 0.05 compared to HgCl_2_-treated group.

It is known that B-cell leukemia/lymphoma-2 (Bcl-2) family proteins are upstream regulators of mitochondrial events and play critical roles in mitochondria-mediated apoptosis. The Bcl-associated X protein (Bax)/Bcl-2 ratio is of particular interest because of its significance in mitochondria-mediated cellular functions. HgCl_2_ caused a decrease and increase in expression of Bcl-2 and Bax, respectively. This led to an increased ratio of Bax to Bcl-2 and, subsequently, to cell apoptosis through a mitochondria-dependent pathway (Figure 8).

### Effects of luteolin on P53, nuclear factor-κB (NF-κB) and tumor necrosis factor α (TNF-α) protein levels

**Figure 9:**
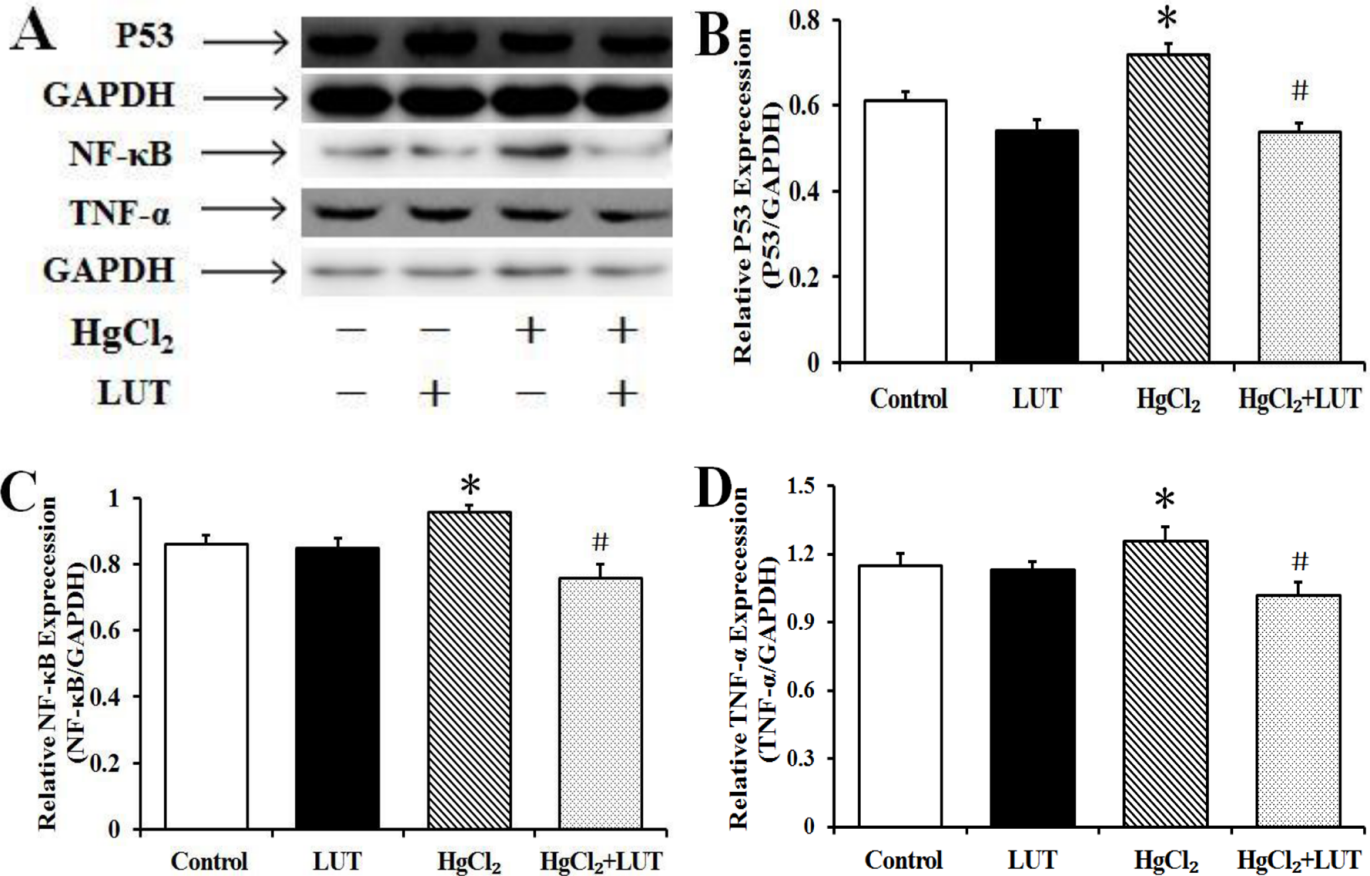
(**A**) Effects of luteolin on P53, NF-κB and TNF-α activaties induced by HgCl_2_ in the liver. These activities were detected by immunoblotting and GAPDH was used as loading control. (**B**, **C** and **D**) Quantifed protein levels were shown. Data are expressed as means ± SEM (*n* = 4). *P < 0.05 compared to the control group; ^#^*P* < 0.05 compared to HgCl_2_-treated group.

P53 proteins levels were increased by HgCl_2_ and this effect was suppressed by luteolin treatment (Figure 9A and 9B). In HgCl_2_-treated rats, HgCl_2_ caused an increase in expression of NF-κB than in the control group. Conversely, treatment with luteolin decrease NF-κB protein expression (Figure 9C). In addition, TNF-α expression followed the same trend as NF-κB (Figure 9D).

### Mercury accumulation in the livers of rats

**Figure 10:**
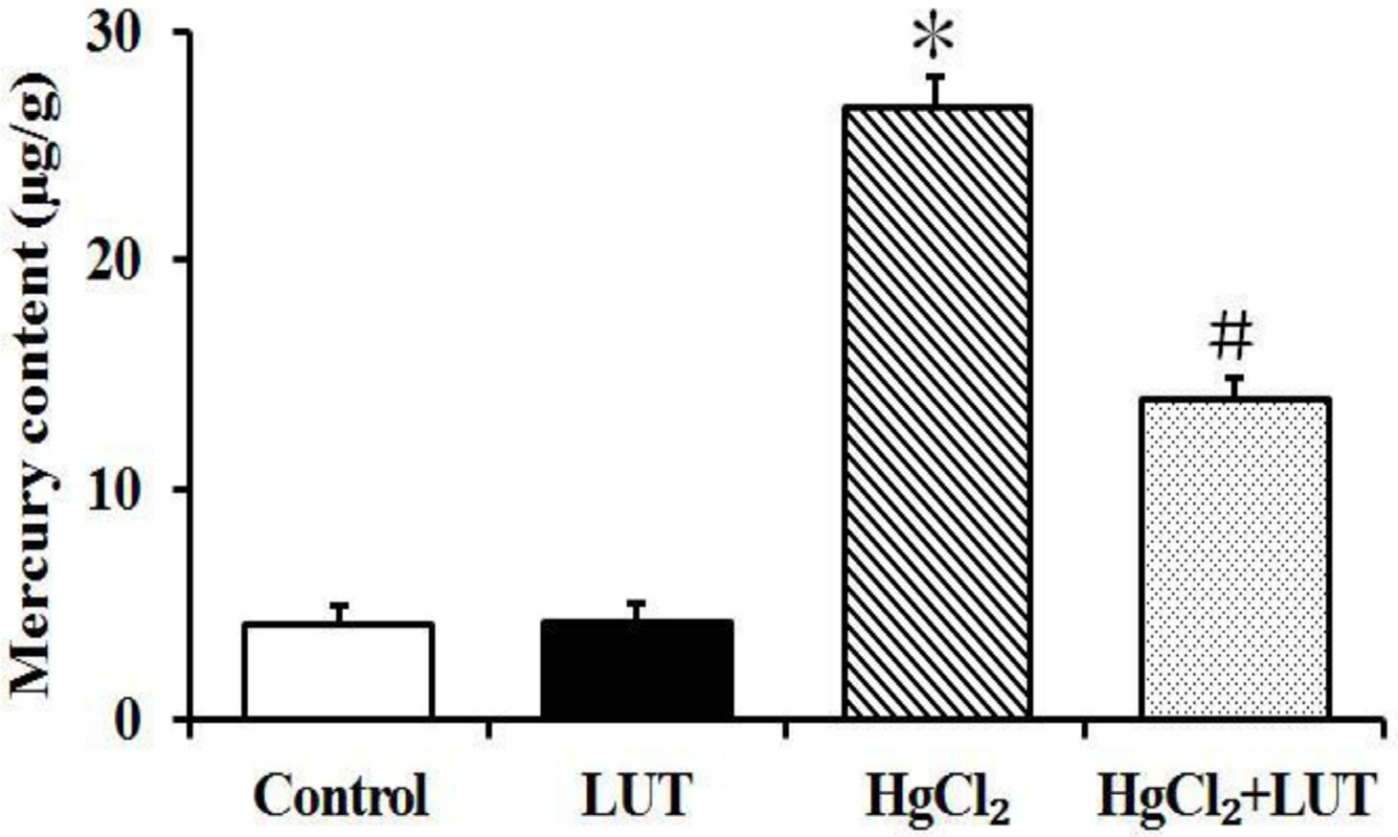
Mercury content analysis was done in the liver and mercury exposed rats as described in materials and methods Tissues were digested in HNO_3_, followed by analysis by atomic fluorescence spectrometry. Data are expressed as means ± SEM (*n* = 8). *P < 0.05 compared to the control group; ^#^*P* < 0.05 compared to HgCl_2_-treated group.

Effects of luteolin on mercury accumulation in the liver were shown in Figure 10. Treatment with HgCl_2_ alone led to significantly increased mercury concentrations, compared with the control group. Treatment with luteolin to mercury exposure significantly decreased mercury accumulation in the liver tissue.

## DISCUSSION

In vertebrate organisms, erythrocytes are the principal means for delivering oxygen (O_2_) to body tissues, via blood flowing through the circulatory system. Hemoglobin is the iron-containing oxygen-transport metalloprotein in red blood cells and the hematocrit is the volume percentage (%) of erythrocyte in blood [16]. We observed decreases in these two indexes, indicating that HgCl_2_ interrupted the supply of O_2_ to the body, inducing anemia in the rats. There was an increase in leukocyte counts in rats exposed to HgCl_2_ and administration of luteolin was decrease. Platelet counts in rats exposed to HgCl_2_ were decreased significantly compared with the control group. However, in this study, mercury exposure had no significant effects on MCV, MCHC or MCH values. This might have been because of the dose and duration of administration used in our exposure protocol.

The amount of a metal accumulated at the target organ has been described as causing its toxicity [17]. According to Flora *et al*. [18], mercury toxicity can cause free radical generation in many tissues, especially the liver. AST and ALT are important markers for hepatocellular damage, as confirmed by Abdel-Moneim *et al*. [19]. In our study, we showed that treatment with luteolin significantly decreased mercury accumulation in the liver tissues. Increased serum AST and ALT activities have been attributed to the damage of liver structural. The increased of AST and ALT were confirmed by histological examination. The changes observed primarily included hepatocellular necrosis or apoptosis, inflammatory cell infiltration and other histological manifestations, all effects consistent with other reports [20]. Therefore, our findings indicated that the presence of luteolin led to improvements in HgCl_2_-induced organ dysfunction.

Oxidative stress is induced when cellular levels of ROS exceed antioxidant response capacity. This results in perturbation of the cellular redox state by producing peroxides and free radicals that damage lipids, DNA and protein [21–23]. We observed increased ROS production *in vitro* in hepatocytes exposed to HgCl_2_. High levels of ROS are known to induce lipid peroxidation, detectable by increased MDA levels [24]. HgCl_2_ manifests its toxicity by increasing ROS and binding to thiol groups in several proteins and peptides, including GSH and many of the antioxidant enzymes [25]. As shown in Figure 2B and 2C, HgCl_2_ treatment caused decreases in both GSH level and GSH/GSSG ratio. All our data indicated that luteolin eliminated ROS and prevented the HgCl_2_-induced decrease in antioxidant defenses.

Inflammation is often associated with an over-production of ROS and is considered important for HgCl_2_-induced liver injury. Inflammation is defined as a defense mechanism involving activation, by pro-inflammatory mediators, of NF-κB as a primary event. Previous research suggested that TNF-α was crucial in cigarette smoke-induced inflammation [26]. In our study, NF-κB activation and significant TNF-α upregulation in the livers of rats treated with HgCl_2_ were substantially attenuated by luteolin (Figure 9). This suggested that attenuation of HgCl_2_-induced liver injury by luteolin was related to its anti-inflammatory effect.

The Bcl-2 family of intracellular proteins are considered key regulators of apoptosis [27]. Our immunoblotting studies demonstrated that HgCl_2_ downregulated antiapoptotic (Bcl-2 and Bcl-xL) and upregulated proapoptotic (Bax) family members, increasing the Bax/Bcl-2 ratio in the liver tissue. However, luteolin treatment prevented these changes. In addition, the apoptotic index and cell viability of hepatocytes were determined with TUNEL and CCK-8 assays, respectively (Figure 4 and Figure 5A), with results consistent with observed effects on Bcl-2 family proteins. These findings suggested that luteolin was pivotal in regulating apoptosis, while inhibiting free radical production and oxidative stress-induced cell death.

The mitochondria are crucial in apoptosis. Cytoplasmic P53 was suggested as activating mitochondrial-mediated apoptosis [28] and increased nuclear P53 was shown to indicate decreased apoptosis [29]. FoxO family transcription factors, particularly FoxO3a, are commonly associated with induction of autophagy-related genes [30]. In previous reports, phosphorylated FoxO3a promoted p53 translocation to the cytoplasm and FoxO3a phosphorylated at Thr32 promoted FoxO3a export from the nucleus [31]. We observed that luteolin enhanced mRNA levels of FoxO3a transcription factors. Thus, we speculated that luteolin affected regulation of P53 localization by FoxO3a. However, further investigation is needed to elucidate the specific mechanism.

Nrf2, a factor sensing the presence of oxidative stress, regulates transcription of genes encoding for cytoprotective enzymes and other proteins crucial for maintaining cellular homeostasis. Under physiological conditions, the Nrf2 inhibitor, Keap-1, binds and retains Nrf2 in the cytoplasm [32]. Nrf2 is inhibited by binding to its negative regulator Keap1, thus modulating its activity [32]. During oxidative stress, Nrf2 dissociates from Keap1, translocates to the nucleus and activates ARE-dependent gene expression, including transcription of such target genes as NQO-1 and HO-1 [33–34]. Increased cell death caused by HgCl_2_ might be attributed to insufficient ROS removal because of a failure in Nrf2 activation. However, we found that luteolin could activate this antioxidant pathway, affecting both Nrf2 protein levels and expression of its target proteins. Therefore, our results suggested that Nrf2 activation was required for protection of the liver, by luteolin, from HgCl_2_-mediated cell death.

**Figure 11:**
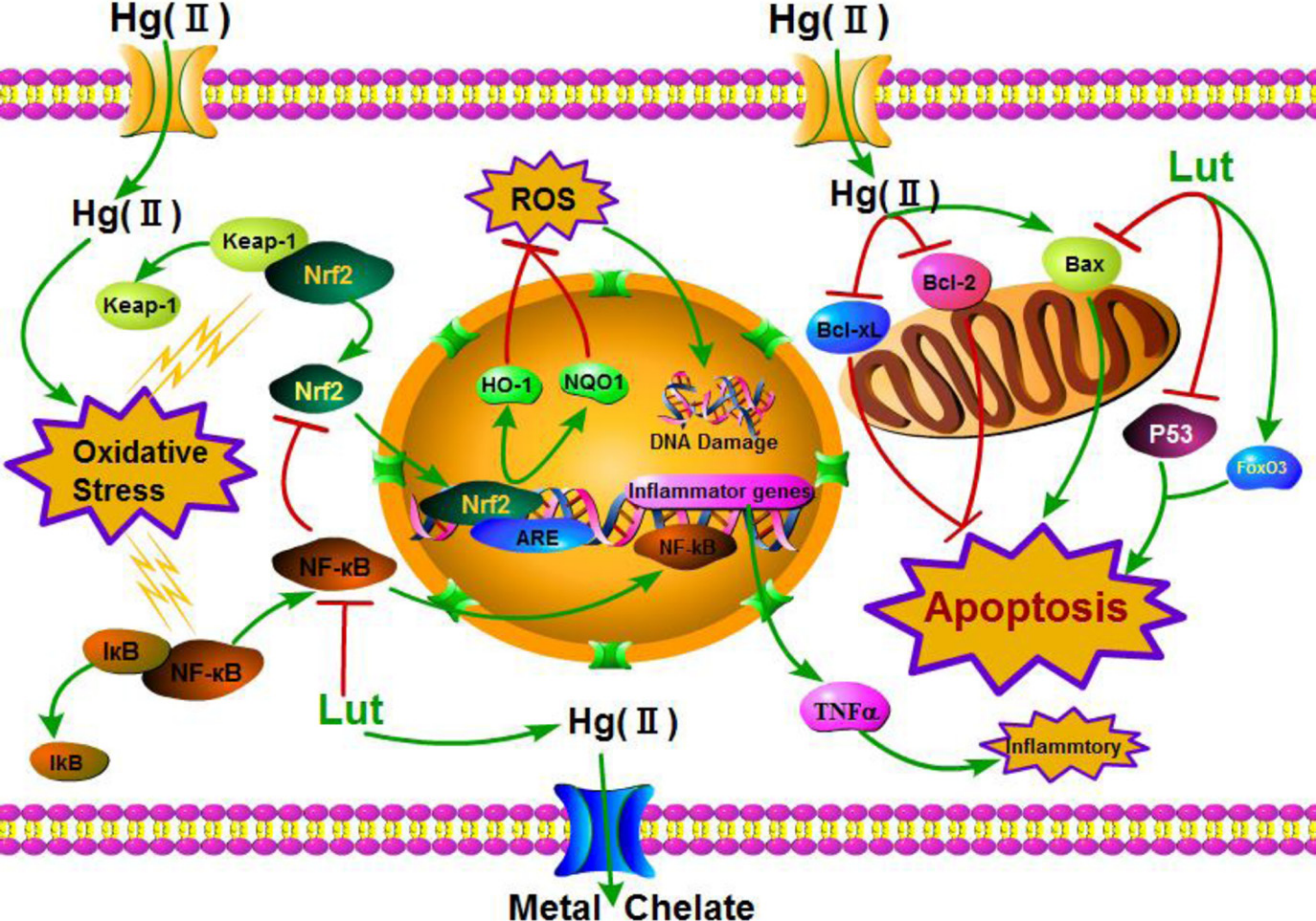
Summary indicating the mechanisms of luteolin attenuated liver injury induced by HgCl_2_ Hg(II) promoted ROS formation in cytoplasm inducing oxidative stress, finally led to apoptosis. However, luteolin triggered the activation of NF-κB/Nrf2/P53 signaling pathway and prevented liver injury induced by Hg(II). Green line denotes stimulatory or inhibitory effect of luteolin, and red line denotes inhibitory effect.

In conclusion, both *in vitro* and rat models, our study demonstrated that dietary luteolin attenuated chronic liver injury induced by HgCl_2_, through regulation of the Nrf2/NF-κB/P53 signaling pathway (Figure 11). Therefore, luteolin administration may be a novel therapeutic approach for treating inorganic mercury poisoning, although optimizing timing to dose is a key question that must be explored in future studies.

## MATERIALS AND METHODS

### Animals

All procedures used in this study were approved by the Institutional Animal Care and Use Committee of Northeast Agricultural University. Twenty-eight 6–8 weeks old male Wistar rats come from the Experimental Animal Centre of Harbin Veterinary Research Institute of Chinese Academy of Agricultural Sciences (Harbin, China) with the weight ranging from 110 to 130 g. The rats were housed individually in an environmentally controlled room maintained at 24–26°C, with a 12 h interval light-dark cycle for 1 week. All rats were provided with water and a balanced diet *ad libitum*.

### Experimental protocol

Wistar rats were randomly divided into the following 4 groups: control, Lut (purity > 98%, Xi’an Weiao, Shanxi, China), HgCl_2_ (Beijing Chemical Factory, China) and HgCl_2_ + Lut groups, 7 rats each group. The rats received 80 mg/L HgCl_2_ in their drinking water for 8 weeks. Freshly made HgCl_2_-containing drinking water was provided every day. Luteolin was gavaged to rats once daily for the last 2 weeks at dose levels of 80 mg/kg in 1% DMSO, and the daily application volumes of luteolin were calculated based on the most recently recorded body weight of the individual animal. Twenty-four hours after the last feeding, surgery was performed on all rats under anesthesia to obtain the blood and liver tissue samples. For histological and TUNEL analysis, a portion of tissues was immersed in 4% paraformaldehyde overnight. The rest of samples were immediately frozen in liquid nitrogen and stored at −80°C until use.

### Biochemical analysis

Blood samples were collected from the abdominal aorta into evacuated tubes and immediately centrifuged at 4,000 rpm for 10 min at 4°C to separate serum. Serum ALT and AST activities were determined to assess the liver function by an Automatic Biochemical Analyzer (UniCel DxC800, Beckman Coulter, USA).

### Determination of parameters associated with oxidative stress in the liver tissues

Liver tissues were rapidly excised and homogenized in phosphate-buffered saline (pH 7.4, w/v; 1 g tissue with 9 mL PBS) with an Ultra-Turrax T25 Homogenizer. After centrifugation at 4,000 rpm for 10 min at 4°C, the liver samples were used to analyze parameters related to oxidative stress including MDA, GSH and GSSG. These parameters were determined by commercial kits (Jiancheng Bioengineering Institute, Nanjing, China) according to the manufacturer's instructions. The tests were conducted by a spectrophotometer (T60 UV–VIS; Beijing Purkinje General Instruments, Beijing, China).

### Histopathological analysis

For the histological analysis, the liver tissues were removed from 10% formalin, then embedded in paraffin. Sections with 3 μm thick midsections of livers were excised from each group and stained with H&E. After finishing the routine pathology of H&E staining, the slides were evaluated by the specific histological alteration of livers under a light microscope (BX-FM, Olympus Corp, Tokyo, Japan).

### TUNEL assay

The apoptotic cells were determined with a TUNEL detection kit (Kaiji, Nanjing, China). For TUNEL assay, coverslips were dealt with 1% Triton X-100/PBS, and blocked with 3% H_2_O_2_/methanol for 15 min, then washed with PBS followed by digesting for 15 min in proteinase K. After being washed with PBS, sections were separately incubated with the Streptavidin-FITC (100 μL) and DAPI (100 μL) label solution in the dark for 1 h at 37°C. The sections displaying green staining within the nucleus were counted as apoptotic cells under a fluorescence microscope.

### Hepatocytes culture

Hepatocytes were isolated by a modification of the two-step collagenase perfusion method described by Zhang et al. (2012) [35]. Hepatocytes from the rat were grown in Dulbecco's modified Eagle's medium (DMEM) supplemented with 10% FBS, penicillin (100 IU/mL) and streptomycin (100 mg/mL) and maintained at 37°C in a humidified atmosphere in a 5% CO_2_ incubator.

### Hepatocytes viability assay

Hepatocytes viability was assessed by the cell counting kit-8 (CCK-8 kit, Beyotime biotechnology, China), according to standard protocols. Briefly, 5 × 10^4^ hepatocytes were seeded in a 96-well plate containing DMEM supplemented with 10% FBS, grown at 37°C for 24 h.

*In vitro* experiments, hepatocytes were divided into 4 groups: control, luteolin-treated (20 μM), HgCl_2_-treated (5 μM) and HgCl_2_+luteolin-treated. Hepatocytes were pre-incubated with luteolin 24 h before HgCl_2_ administration. After that, the original medium was replaced by 100 μL 10% FBS DMEM medium contain 10 μL CCK-8 solution, followed by incubation at 37°C for 2 h. The absorbance was finally determined at 450 nm with a microplate reader.

### Determination of intracellular levels of ROS

Intracellular ROS levels were detected with a ROS assay kit (Beyotime biotechnology, China). DCFH-DA was deacetylated intracellularly by nonspecific esterase, which was further oxidized by ROS to the fluorescent compound 2,7-dichlorofluorescein (DCF). After treatment with HgCl_2_ and luteolin by means of hepatocytes viability assay, hepatocytes were pre-incubated with DCFH-DA at a concentration of 10 μM. After 20 min, DCFH fluorescence was measured at an excitation wavelength of 488 nm and an emission wavelength of 525 nm with a Nikon TE2000 inverted microscope system.

### Real-time PCR analysis

Total RNA was extracted from the frozen livers with Trizol reagent (Invitrogen, US). The total RNA concentration and purities were determined at 260 nm. The target genes were quantified by SYBR^®^ Premix Ex Taq^™^ kit (Life Technologies, USA) according to the manufacturer's instructions. Using the 2^−ΔΔCt^ method, the results were presented as the fold change in the target gene expression normalized to the endogenous reference gene (β-action) and relative to the untreated control. The primer sequences used in this study were shown in the Table 2.

**Table 2:** Primers used for real-time PCR1

Gene	5′— 3′ Primer sequence	Product size (bp)
**FoxO3 - F**	GAGTCCATCATCCGTAGC	438
**FoxO3 - R**	GAGGGTCAAAGGAAACAA	
**β-actin - F**	GAGAGGGAAATCGTGCGTGAC	452
**β-actin - R**	CATCTGCTGGAAGGTGGACA	

### Western blot analysis

Total protein concentration in the supernatant was determined with a bicinchoninic acid assay (Beyotime biotechnology, China). The equal amounts of protein were separated by SDS-PAGE on 8–12% Bis-Tris gels, transferred onto PVDF membranes. Primary antibodies against each protein were from Santa Cruz Biotechnology (Santa Cruz, CA, USA). Antibodies to GAPDH (Xianzhi, Hangzhou, China) were used as a standard control. The membrane was detected with the Imager Amersham 600 chemiluminescence system (General Electric Company, Fairfield, CT, USA).

### Determination of mercury accumulation in the liver

Briefly, approximately 0.5 g liver tissues were digested with HNO_3_–H_2_SO_4_ solution. After aspiration and tomization of the sample, a light beam from a hollow cathode lamp was directed through the flame into a monochromator and onto a detector that measured the amount of light absorbed. The mercury content of liver tissues were analyzed by atomic fluorescence spectrometry (AFS-930; Beijing Jitian Instrument Company, Beijing, China).

### Statistical analysis

Statistical analysis was performed by the SPSS 19.0 (SPSS, Chicago, IL, USA). All data were expressed as mean ± SEM. The data in this report were evaluated by one-way ANOVA or Student's t Test. In all cases, *P* < 0.05 were considered to be significant for comparison among different groups.

## Abbreviations

ALT: alanine aminotransferase
AST: aspartate transaminase
Bax: Bcl-associated X protein
Bcl-2: B-cell leukemia/lymphoma-2
Bcl-xL: Bcl-2-related protein long form of Bcl-x
DCF: 2,7-dichlorofluorescein
DMEM: dulbecco's modified Eagle's medium
DMPS: 2,3-dimercaptopropanesulfonic acid
DMSA: meso-2,3-dimercaptosuccinic acid
FoxO3: forkhead box O3
GSH: reduced glutathione
GSSG: glutathione disulfide
H&E: hematoxylin and eosin
HgCl_2_: mercuric chloride
HO-1: Heme oxygenase-1
Lut: luteolin
MCH: mean corpuscular hemoglobin
MCHC: mean corpuscular hemoglobin concentration
MCV: Mean corpuscular volume
MDA: malondialdehyde
NF-κB: nuclear factor-κB
NQO1: NAD(P)H quinone oxidoreductase 1
Nrf2: nuclear factor-erythroid-2-related factor 2
O_2_: oxygen
ROS: reactive oxygen species
TNF-α: tumor necrosis factor α
TUNEL: terminal deoxynucleotidyl transferase-mediated dUTP nick end labeling
